# Fire-Retardant Flexible Foamed Polyurethane (PU)-Based Composites: Armed and Charmed Ground Tire Rubber (GTR) Particles

**DOI:** 10.3390/polym16050656

**Published:** 2024-02-28

**Authors:** Paulina Kosmela, Kamila Sałasińska, Daria Kowalkowska-Zedler, Mateusz Barczewski, Adam Piasecki, Mohammad Reza Saeb, Aleksander Hejna

**Affiliations:** 1 Department of Polymer Technology, Gdańsk University of Technology, Narutowicza 11/12, 80-233 Gdańsk, Poland; paulina.kosmela@pg.edu.pl; 2 Faculty of Materials Science and Engineering, Warsaw University of Technology, Wołoska 141, 02-507 Warsaw, Poland; kamila.salasinska@pw.edu.pl; 3 Department of Inorganic Chemistry, Gdańsk University of Technology, Narutowicza 11/12, 80-233 Gdańsk, Poland; daria.zedler@pg.edu.pl; 4 Institute of Materials Technology, Poznan University of Technology, Piotrowo 3, 61-138 Poznań, Poland; mateusz.barczewski@put.poznan.pl; 5 Institute of Materials Engineering, Poznan University of Technology, Jana Pawła II 24, 60-965 Poznan, Poland; adam.piasecki@put.poznan.pl; 6 Department of Pharmaceutical Chemistry, Medical University of Gdańsk, J. Hallera 107, 80-416 Gdańsk, Poland; mrsaeb2008@gmail.com

**Keywords:** polyurethane foam, composites, ground tire rubber, filler modification, flame retardancy, flammability

## Abstract

Inadequate fire resistance of polymers raises questions about their advanced applications. Flexible polyurethane (PU) foams have myriad applications but inherently suffer from very high flammability. Because of the dependency of the ultimate properties (mechanical and damping performance) of PU foams on their cellular structure, reinforcement of PU with additives brings about further concerns. Though they are highly flammable and known for their environmental consequences, rubber wastes are desired from a circularity standpoint, which can also improve the mechanical properties of PU foams. In this work, melamine cyanurate (MC), melamine polyphosphate (MPP), and ammonium polyphosphate (APP) are used as well-known flame retardants (FRs) to develop highly fire-retardant ground tire rubber (GTR) particles for flexible PU foams. Analysis of the burning behavior of the resulting PU/GTR composites revealed that the armed GTR particles endowed PU with reduced flammability expressed by over 30% increase in limiting oxygen index, 50% drop in peak heat release rate, as well as reduced smoke generation. The *Flame Retardancy Index* (*FRI*) was used to classify and label PU/GTR composites such that the amount of GTR was found to be more important than that of FR type. The wide range of *FRI* (0.94–7.56), taking *Poor* to *Good* performance labels, was indicative of the sensitivity of flame retardancy to the hybridization of FR with GTR components, a feature of practicality. The results are promising for fire protection requirements in buildings; however, the flammability reduction was achieved at the expense of mechanical and thermal insulation performance.

## 1. Introduction

Both market reports [1,2] and scientific publications [3,4,5] point to the rapid development of the construction and building sector in the following years, which can be attributed to the economic growth of the society and stronger-than-ever economic migration. Simultaneously, law regulations and environmental awareness are pushing all of the industry sectors, including construction and building, towards more sustainable solutions and materials [6,7,8,9,10]. Considering these features, cellular materials, including polyurethanes (PU), pose as auspicious candidates for novel applications in construction and building materials. PU foams have been commonly applied in buildings over the last decades, either as thermal, sound, or mechanical insulation [11,12,13,14]. Conventionally, PUs are produced from petroleum-based materials, but keeping in mind current trends and regulations, they should shift towards recycled, waste-based, or renewable resources, which could enhance their resource efficiency [15,16,17]. The application of such sustainable PU foams, except for the reduced depletion of natural resources, should provide similar or superior performance compared to the conventionally applied materials, e.g., in the case of energy efficiency [18,19]. Extensive efforts have been made over the last years to investigate and develop novel sustainable solutions for PU foams comprehensively. They can be divided into the application of polyols from plant-based materials [20,21,22,23] or industrial residues [24,25,26], the development of non-isocyanate PUs [27,28,29,30], as well as the incorporation of natural [31,32,33,34] or waste-based fillers [35,36,37,38,39], and additives like flame retardants [40,41], which could reduce the utilization of virgin PU and provide novel functionalities in a sustainable manner. 

These directions address different aspects of the sustainability-related challenges of PUs. Avoiding the use of isocyanates is related to their high reactivity and resulting toxicity, which poses threats during manufacturing and decomposition of material. The application of renewable raw materials, either in the form of fillers or during biopolyol production, leads to the depletion of the environment, as it may require additional plant cultivation and often compete with food production [42,43]. On the other hand, the introduction of industrial residues, especially burdensome ones, may not only provide beneficial features to PU foams but also lead to the development of novel management routes for various wastes. Therefore, it should be considered an auspicious approach.

Except for the environmental aspects, the incorporation of waste-based or recycled materials may provide PUs additional features resulting from the structure or performance of virgin materials. Among the potential waste fillers can be mentioned PU foam scraps [44,45,46], waste lignocellulose fillers [47,48], textiles and leather wastes [49,50], and waste polymer or rubber materials [36,51]. The last group of materials is auspicious because it can often take advantage of the excellent performance of primary materials, mainly car tires, which can be applied in the form of ground tire rubber (GTR).

As presented in our previous works on PU/GTR composite foams [52,53], the introduction of waste rubber particles may beneficially affect the cellular structure due to the GTR nucleating activity, yielding a reduction in average cell size. Structural changes may enhance the insulation performance expressed by the decrease in thermal conductivity coefficient, as well as the static and dynamic mechanical performance. Combined with the low cost compared to commercial PU systems [54], these beneficial changes in structure and performance make GTR an auspicious candidate for PU foam filler. 

Nevertheless, despite the undeniable advantages of GTR incorporation, its use also has a second face related to the deepening of one of the biggest shortcomings of PU foams: their flammability. The low fire resistance of porous polyurethane materials results not only from their chemical structure but also from the geometric structure itself: namely, large specific surface area and exposure to the oxidizing atmosphere during combustion. Conventionally, PU foams ignite relatively easily and burn at high rates, yielding the formation of often toxic smoke [55]. Currently, increasingly more attention is being paid to the reduction of flammability of PU materials, often applied as building materials or furniture, which are very close to everyday human life. So far, numerous, often excellent, solutions dealing with the severe flammability of PU foams have been developed and comprehensively described in the literature. The latest advances have been recently summarized in the excellent review works of Morgan [52], Liu et al. [53], Muhammed Raji et al. [54], and Yadav et al. [56]. These works provided numerous methods for fire protection of flexible PU foams, including reactive and additive flame retardants (FRs) and various types of coatings. However, considering the incorporation of GTR, its flammability has to be also considered [57]. Therefore, its application as a PU filler makes the challenge of reducing PU flammability more difficult. Due to the commonness of PU foams in everyday life related to their broad application range, as well as the potential use of PU/GTR composite foams as construction materials, their high flammability needs to be reduced. Reducing the flammability of such complex materials, comprising foamed matrix and solid filler GTR particles, is a great challenge.

So far, the only work reporting investigation on the flammability of PU/GTR foams was published by Ryszkowska et al. [58]. The authors presented the results of limiting oxygen index (LOI) and microcalorimeter combustion for semi-rigid foams containing GTR in the amount of 25 parts per hundred parts of polyol (php). To improve fire resistance, composites were modified with 25 php of expandable graphite, 5 php of organophosphorous FR Fyrol PNX, and a combination of both. Applied modifications enabled an 8–34% increase in LOI, an 8–68% decrease in total heat released, and an up to 78% decrease in maximum heat release rate. The presented results point to the high efficiency of a combination of expandable graphite and organophosphorus FR, but at the same time, highlight that the effect of FRs on the combustion of polymeric materials is a very complex process, which can co-occur according to several mechanisms involving numerous chemical reactions and physical interactions.

Among the available FRs, the most popular, apart from those containing halogens, are aluminum and magnesium hydroxides, followed by compounds containing phosphorus and nitrogen atoms. A significant trend in research on reducing the intensity of the burning process and the emission of toxic fumes from polymer materials is the synergistic effect. For some FRs containing both phosphorus and nitrogen, an increase in the effectiveness corresponds to the sum of individual interactions, prompting researchers to create new substances or FR systems. Currently, intumescent FRs are considered among the most effective, forming a stiff carbonized layer of cellular structure on the surface of the polymer modified with them, which protects the material against the heating of its deeper layers and prevents the exchange of matter between the phases. 

Herein, the presented study evaluated the multithreaded approach to reducing the flammability of flexible foamed PU-based composites by hybridization of GTR particles with solid FRs, ammonium polyphosphate (APP), melamine cyanurate (MC), and melamine polyphosphate (MPP). The presented work examined novel, non-standard applications of these FRs commonly applied for PU flammability reduction on an industrial scale. The application of these FRs aligns with the current halogen-free policy postulated to reduce polymers’ flammability, which is expressed by various law regulations. The impact of applied modifications on the cellular structure, thermal insulation, and mechanical performance, as well as the flammability and thermal stability of PU/GTR composite foams, has been comprehensively assessed.

## 2. Materials and Methods

### 2.1. Materials

Table 1 provides the details on the raw materials applied for the manufacturing of flexible foamed PU/GTR composites with reduced flammability.

### 2.2. Modifications of GTR

**Figure 1:**
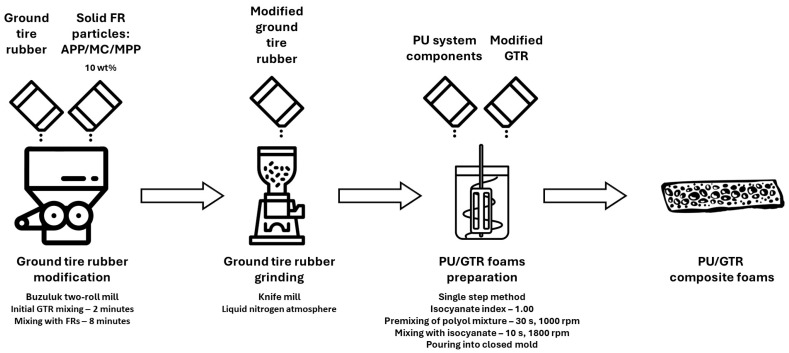
General scheme of sample preparation, including GTR modification and preparation of PU/GTR composite foams.

To reduce the unfavorable impact of GTR on the performance of prepared composites, it was additionally subjected to mechanical modification assisted by 10 wt% of solid particles of FRs—MPP, MC, and APP. Modifications were performed using a two-roll mill model 14201/P2 from Buzuluk (Komarov, Czech Republic). Processing time equaled 10 min and included a 2 min phase of initial mixing of GTR alone to reduce the agglomerates and soften the rubber particles. Further, GTR with FRs was repeatedly passed through rotating rolls of a two-roll mill for 8 min. Afterward, prepared samples were ground using an A11 analytical mill from IKA-Werke GmbH & Co. KG (Staufen im Breisgau, Germany). A general scheme of sample preparation, including GTR modification and preparation of PU/GTR composite foams, is presented in Figure 1. SEM images of modified GTR samples are presented in Figure S1.

### 2.3. Preparation of PU/GTR Composite Foams

PU foams were manufactured on a laboratory scale by a single-step method using an isocyanate index of 1.00. The polyol component, including all raw materials except isocyanate, was mechanically mixed for 30 s at 1000 rpm to ensure homogeneity. Subsequently, the polyol mixture was mixed with isocyanate for 10 s at 1800 rpm, and they were poured into a closed aluminum mold with dimensions of 20 × 10 × 4 cm. After demolding, the samples were conditioned at 60 °C for 12 h and then at room temperature for 24 h. Table 2 provides the details of foam formulations. The formulations and preparation were adjusted to provide a similar apparent density of all samples, which was in the range of 186–190 kg/m^3^. Composites filled with unmodified GTR without additional FRs were named GTR_5_ and GTR_10_, composites containing unmodified GTR and FRs (PLO and EG) were named GTR_5_FR and GTR_10_FR, while composites containing FRs and modified GTR were named GTR_X5_FR and GTR_X10_FR, where X stands for FR used for GTR modifications—MPP, MC, or APP.

### 2.4. Characterization Techniques

A MIRA3 (Brno, Czech Republic) scanning electron microscope (SEM) was used to investigate the microstructure of the obtained foams. During measurements, 5 kV accelerating voltage for cell structure analysis and a secondary electron detector were used. The obtained images of the structures were analyzed using ImageJ v.153i computer software. 

The content of the open cells in the polyurethane foams was assessed by the Anton Paar Ultrapyc 5000 Foam gas pycnometer from (Graz, Austria). The following measurement settings were applied: gas—nitrogen; target pressure—3.0 psi; measurement type—corrected; temperature control—on; target temperature—20.0 °C; flow mode—monolith; cell size—45 cm^3^; preparation mode—flow, 0.5 min. 

The thermal conductivity (λ) coefficient was investigated by means of the Netzsch HFM 446 Lambda plate apparatus (Selb, Germany). The rectangular samples with dimensions of 10 × 10 × 4 mm were measured with a caliper to the nearest 0.1 mm and weighed on an analytical balance to the nearest 0.0001 g. The specimens were tested in the temperature range of 1–19 °C at an average temperature of 10 °C.

The mechanical properties of the novel PUR/GTR composite foams were evaluated by tensile strength according to the PN EN ISO 1798:2009 standard [59]. Specimens with dimensions 10 × 10 × 100 mm were measured using calipers with an accuracy of 0.1 mm. The tensile test was conducted on a Zwick/Roell Z020 universal testing machine (Ulm, Germany) at a cross-head constant speed of 500 mm/min. The series of five samples were tested for each foam. 

The compressive strength of the test specimens was estimated in accordance with PN EN ISO 604:2006 [60]. Cylindrical 20 × 20 mm (height and diameter) specimens were measured with calipers with an accuracy of 0.01 mm. The compression test was carried out on a Zwick/Roell Z020 universal testing machine (Ulm, Germany) at a constant speed of 15%/min until a strain of 70% was reached. Five samples were tested for each foam. 

Thermal stability of the samples was carried out using a Netzsch TG 209 F3 apparatus (Selb, Germany). Composite samples with a 10 ± 0.5 mg mass were placed in a ceramic vessel. The tests were conducted in an inert gas–nitrogen atmosphere in the range from 30 to 800 °C with a heating rate of 10 °C/min. 

Fire behavior measurements of flexible PU/GTR composite foams were carried out using a cone calorimeter as described by ISO 5660 standard [61]. Samples with dimensions 100 × 100 × 40 mm were subjected in a horizontal position, in the presence of a spark from an igniter that initiates burning, to a heat flux of 50 kW/m^2^ produced by a conical electric radiant heater, and changes in the oxygen concentration of the combustion gases were measured. From the changes in oxygen concentration, the intensity of heat release was determined. An optical system with a silicon photodiode and a helium–neon laser delivered a survey of smoke. The parameters obtained during cone calorimetry analysis were applied to calculate the flame retardancy index (*FRI*) of PU/GTR composite foams according to the following Equation (1):

*FRI* = (THR × pHRR/TTI)_Neat material_/(THR × pHRR/TTI)_Flame-retarded material_ (1)

where THR—total heat released, MJ/m^2^; pHRR—peak heat release rate, kW/m^2^; TTI—time to ignition, s. To evaluate the impact of applied FRs on the *FRI*, samples GTR_5_ and GTR_10_ were considered neat materials.

Measurements of the LOI were carried out in accordance with PE-EN ISO 4589-2:2006 [62]. Samples in the shape of a cuboid with dimensions of 100 × 10 × 10 mm were placed in a column to measure the minimum concentration of oxygen in a mixture of oxygen and nitrogen at which the flame is maintained.

## 3. Results and Discussion

### 3.1. Microstructure and Physico-Mechanical Performance of PU/GTR Composite Foams

**Figure 2:**
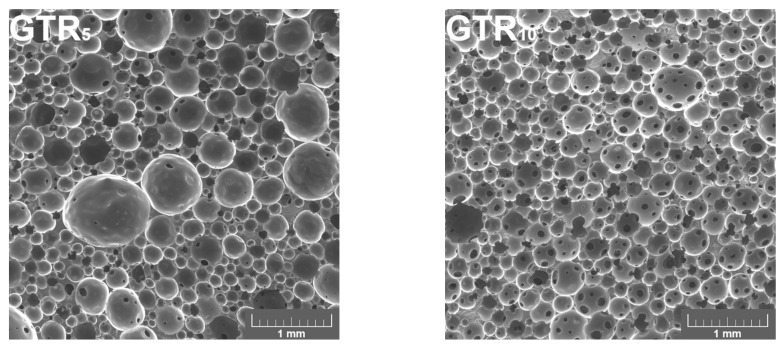

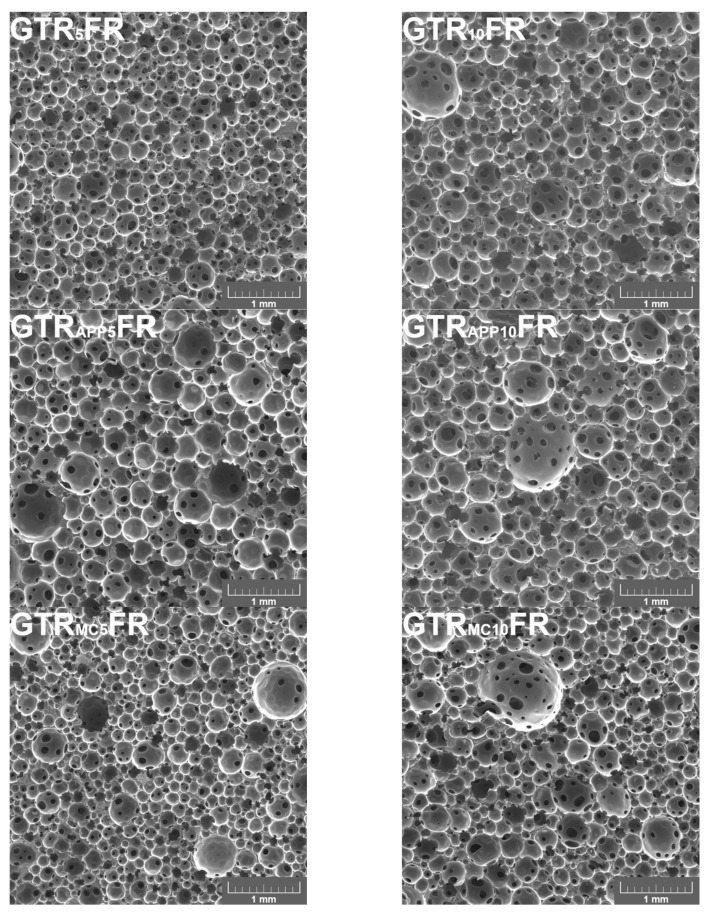

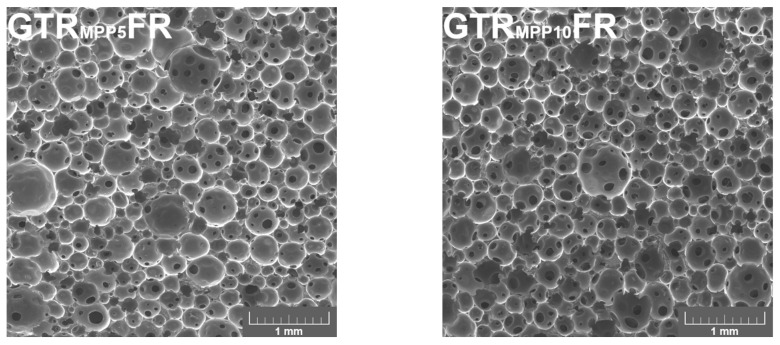
Images of polyurethane (PU)/ground tire rubber (GTR) composite foams’ cellular structure obtained with scanning electron microscopy.

Figure 2 presents the selected SEM images of the cellular structure of PU/GTR composite foams. Quantitative parameters derived from the structure evaluation are presented in Table 3, along with the physico-mechanical properties of analyzed samples.

The presented SEM images indicate that the loading of GTR and the applied FRs showed a noticeable impact on the cell size and heterogeneity of cellular structures. For unmodified GTR particles, the increasing filler loading resulted in a finer cellular structure, confirming our previous results [53] and other works pointing to the nucleating activity of fillers in PU foams [63,64]. Such an effect was also associated with the lower aspect ratio of cells and increased roundness, which indicates that cells were more similar to a perfect circle.

The incorporation of FRs, either as GTR modifiers or additives to formulations, also affected the cellular structure. For the GTR_5_FR sample, the beneficial cell size reduction was noted, which could be attributed to the EG nucleating activity suggested by literature works [65,66]. Among the samples containing modified GTR particles, the finest cell size was noted for the application of MC, which can be related to its lowest particle size (D_50_ below 8 µm according to the producer). Nevertheless, for all of the applied modifiers, the cellular structures are relatively similar, which can be related to the low loading of loose FR particles, which have been rather compressed onto the GTR surface during modification with two roll mill.

Apart from the cell size, another important parameter of cellular structure is related to the characteristics of cells and their open or closed character, which affects the rate of gas and heat exchange within the foam, as well as between the foam and surrounding. The lowest content of open cells was noted for GTR_5_ and GTR_10_ samples, which aligned with the lowest λ coefficients among the analyzed samples. Lower open cell content reduces the convective heat transfer inside cellular materials, enhancing their insulation performance. On the other hand, modification of GTR particles and introduction of additional FRs slightly increased the open cell content and λ coefficients. Nevertheless, obtained values should satisfy the requirements for floor underlays as potential applications of developed PU/GTR composite foams [67].

Table 3 also presents details on the tensile performance of prepared PU/GTR composite foams depending on the applied modifications. Significantly, the highest values of tensile strength exceeding 200 kPa were noted for unmodified PU/GTR composites, matching the values reported in our previous works for similar GTR loadings [68]. At the same time, the highest values of elongation at break were noted, which, however, decreased with GTR content due to increased heterogeneity of structure. The incorporation of FRs caused a noticeable reduction in mechanical parameters, which can be attributed to the plasticizing effect of TCPP, which has been repeatedly reported in the literature [69,70]. The presence of TCPP weakens the interactions between PU macromolecules, affecting the mechanical performance of the final material. Moreover, literature reported the adverse impact of EG on PU foams’ mechanical performance resulting from its particle size, implicating the placement of particles between the cell walls rather than in struts [71]. Therefore, the movements of PU macromolecules were restricted during strain, which limited composites’ elongation at break. The introduction of additional FRs as GTR modifiers enhanced the mechanical performance of composites, which could be associated with the characteristics of the GTR modification process. The application of two roll mill involves significant shear forces acting on the material during mixing, which results in the compressing of solid particles onto GTR and the development of a specific surface area, which further strengthens interfacial interactions with the PU matrix [72]. A similar effect was observed in our previous work dealing with the application of GTR modified with zinc borate particles in a twin-screw extruder [68]. The application of APP resulted in superior tensile performance over MC and MPP, which aligns with the literature reports pointing to the increased *FRI*ability of PU cellular structure due to the application of melamine FRs, especially MC [73,74]. Konig et al. [75] and Kageoka et al. [76] also reported a decrease in elongation at break after the addition of melamine-based FRs to flexible PU foams related to the limited elasticity of composite material and inhibition of complete elongation prior to structural rupture.

### 3.2. Thermal Stability of PU/GTR Composite Foams

**Figure 3:**
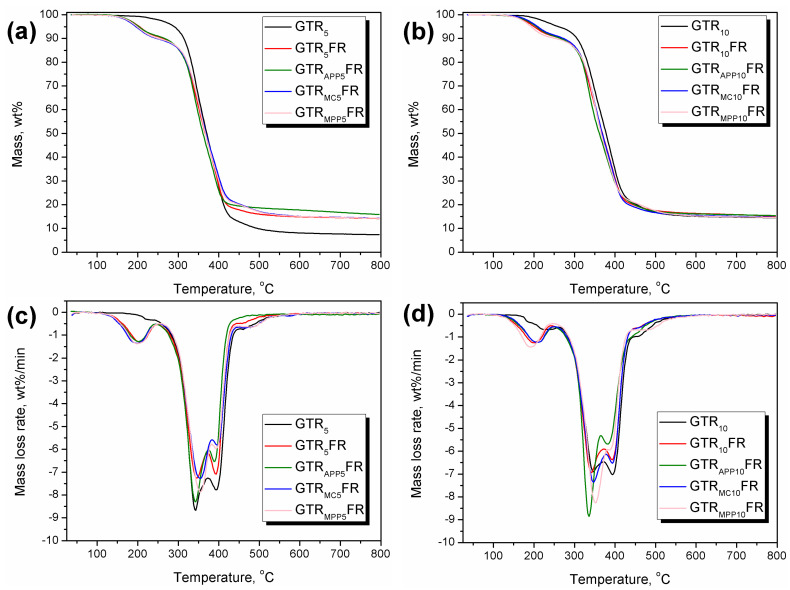
Plots of (**a**,**b**) mass loss curves and (**c**,**d**) differential thermogravimetric curves for PU/GTR composite foams.

The content of GTR in prepared composites, as well as the applied modifications aimed at reducing their flammability, significantly affected the thermal degradation course and rate, which is commonly known for PU materials [55]. Thermal decomposition is critical for the flammability of PU foams because it yields the release of low-molecular-weight molecules, which may diffuse into the flame zone and form a flammable mixture in combination with air [55]. The heat generated during burning accelerates the thermal decomposition, sustaining the combustion cycle. However, the literature indicates the reduction in thermal stability resulting from the application of organophosphorus FRs, despite their beneficial impact on flammability reduction [77,78,79], which is associated with the volatiles release accompanying the protective char layer formation. Therefore, the thermal stability of FR-modified materials should always be evaluated with a profound understanding of the FR mode of action. Figure 3 provides detailed data obtained from TGA analysis of PU/GTR composite foams.

All of the prepared composite foams showed a similar course of thermal decomposition, with the initial degradation step, whose magnitude depended on applied formulation, and the second main step, followed by the transient char degradation. Significant differences have been noted between the thermal stability of composite foams with varying GTR content, which can be attributed to the generation of additional hard segments in PU materials due to the reaction between isocyanate and functional groups present on the GTR surface [68]. Hard segments, comprising urethane, allophanate, and biuret groups, show inferior thermal stability than the long macromolecular chains of polyols [55]. Therefore, the GTR_10_ sample showed a more pronounced initial decomposition step than the GTR_5_. In the case of FR-modified foams, irrespective of the applied formulation and method of FR incorporation, the first step was more significant due to the thermal decomposition of TCPP applied as a GTR modifier. Such a phenomenon was attributed to the low decomposition and volatilization temperature of TCPP, which is also noted in other works [80,81]. The introduction of additional FRs hardly affected the magnitude of the first degradation step due to their higher stability compared to liquid organophosphorus FRs.

The second, most notable decomposition step was attributed to the degradation of PU soft segments, GTR particles, and applied FRs. Soft segments of PU typically decompose between 300 and 420 °C, depending on the applied polyols [82]. Introduced GTR particles originated from post-consumer car tires and consisted of two types of rubber, natural and styrene–butadiene, whose maximum decomposition rate is typically around 370 and 430 °C, respectively [83]. Considering applied FRs, the APP typically shows two-step degradation. The step associated with the evolution of ammonia and water as gaseous products occurs typically between 200 and 400 °C, with a maximum rate of around 328 °C, while above 500 °C the ultraphosphate structures generated in the previous step are decomposed [84,85]. However, APP decomposition starts at lower temperatures, since around 200 °C, it changes its crystalline form along with the partial NH_3_ release. The initial APP degradation overlapped with the decomposition of PU hard segments, which, as mentioned above, despite the flexible character of foams, might have been present at the PU/GTR interface.

Contrary to APP, MC is characterized by single-step decomposition, with an onset around 325 °C and a maximum rate at 405 °C [86], which overlaps with the decomposition of PU soft segments and GTR components. The degradation onset of MPP, similar to MC, exceeds 300 °C, even 350 °C, as reported in publications [87,88]. This course is associated with the sublimation of melamine. In the presented case, it overlaps with the PU soft segments’ and GTR degradation.

The last minor decomposition step results from the degradation of transient char and is typically noted for PU materials.

### 3.3. Flammability of PU/GTR Composite Foams

The LOI is one of the most frequently used tests to evaluate the flammability of polymers. Materials with an LOI of less than 22% *v*/*v*, such as PU with unmodified GTR, are considered flammable. The use of FRs increased the LOI, so they were classified into the group of flame-retarded materials, while in the case of GTR_5_FR, GTR_MC5_FR, and GTR_APP5_FR, even to self-extinguishing (above 28% *v*/*v*). The obtained results suggest that the ignitability of the material increases with increasing the GTR share (Table 4).

**Figure 4:**
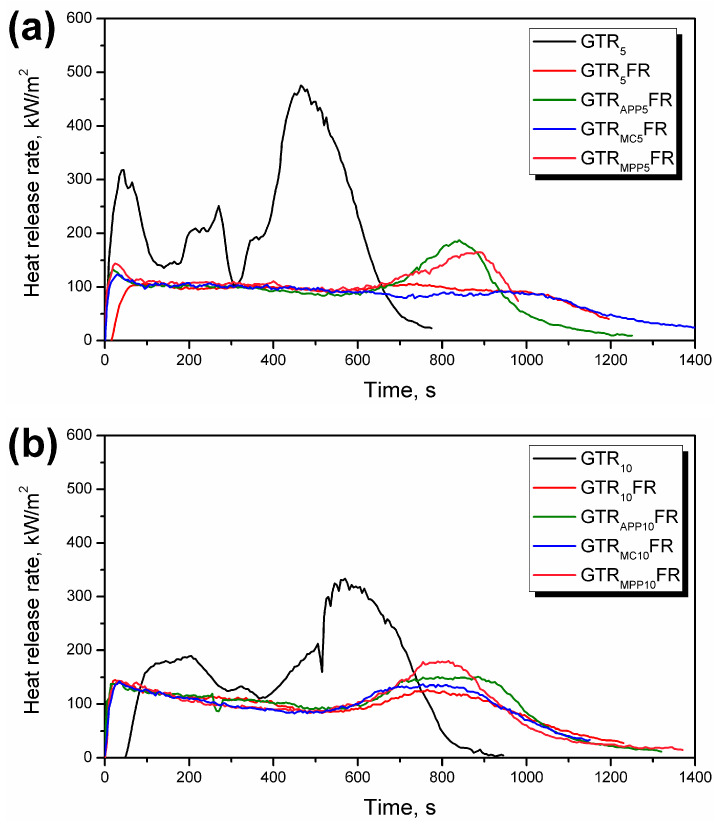
Heat release rate over time during cone calorimetry tests of PU/GTR composite foams containing (**a**) 5 wt% and (**b**) 10 wt% of as-received or modified GTR.

**Figure 5:**
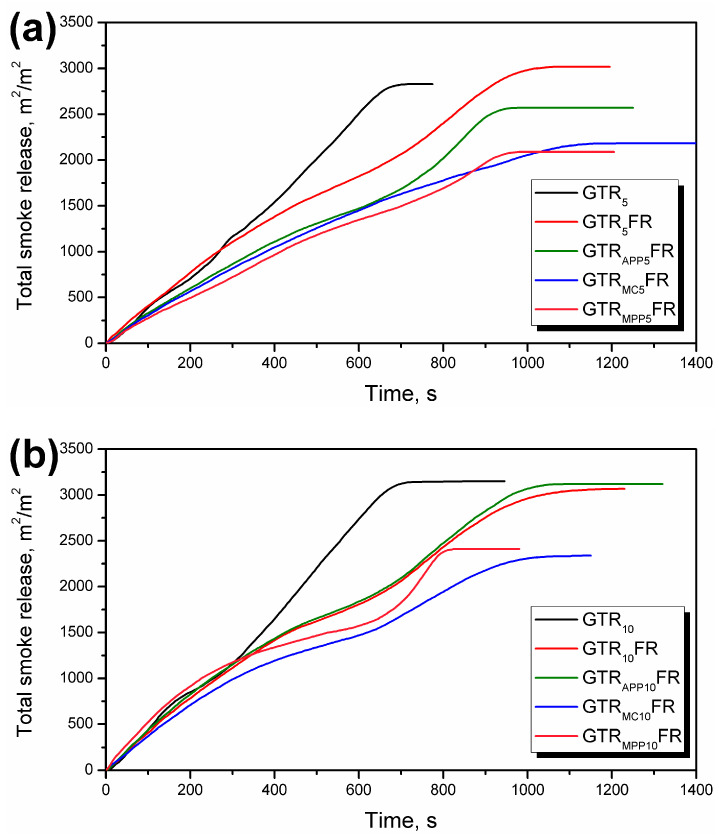
Total smoke release over time during cone calorimetry tests of PU/GTR composite foams containing (**a**) 5 wt% and (**b**) 10 wt% of as-received or modified GTR.

Although LOI provides a measure of flammability required for mass production in the industry, the burning behavior of polymer composites can merely be expected from cone calorimetry [89,90]. The impact of unmodified GTR, unmodified GTR, and FRs, as well as modified GTR and FRs, on PU foam’s flammability performances were also assessed via cone calorimetry measurement. A cone calorimeter is a bench-scale fire testing equipment that allows hazard analysis involving both heat release and fumes emission [91]. The heat release rate (HRR) and total smoke release (TSR) curves are shown in Figure 4 and Figure 5, while detailed data obtained from tests, such as time to ignition (TTI), peak HRR (pHRR), maximum average rate of heat emission (MARHE), total heat release (THR), effective heat of combustion (EHC), and specific extinction area (SEA), are summarized in Table 4.

PUs with unmodified GTR exhibited three peaks, and the last one, equal to 461 kW/m^2^ and 368 kW/m^2^ for GTR_5_ and GTR_10_, respectively, yielded the maximum pHRR. The use of FRs flattened the curves and reduced the number of peaks. The samples showed a rise at the beginning and end of the test, which may have been caused by insufficient strength and cracking of the char. The second peak is also observed in the case of the non-charring materials, resulting from an increase in the effective pyrolysis [92]; however, this mechanism of action is much less probable in the case of investigated materials. EG works mainly in the solid phase and creates a protective layer on the surface of samples caused by the reaction of sulfuric acid with graphite flakes, which expands their volume by about 100 times [93]. The protective effect is based on the limited heat and mass transfer inhibiting the fire spread [94]. Its efficiency can be confirmed by the significantly increased values of char yield provided in Table 4. In turn, halogen-containing fire retardants, including TCPP as a chlorinated phosphoric ester, use chemical interference with the radical chain mechanisms in the gas phase [95]. Phosphorus FRs volatilize into the gas phase and act as scavengers of radicals even more effectively than chlorine-based FRs or remain in the solid phase and promote char formation as APP and MPP [96]. Using FRs with different mechanisms of action in the polymer and in the filler may contribute to increasing their effectiveness through a synergistic effect [97]. The synergistic effect of EG and organophosphorus FRs has been repeatedly reported in the literature [98,99,100]. Authors ascribed the flammability reduction to the combination of a “worm-like” structure formed during EG expansion and the formation of a highly viscous layer originating from phosphorous-catalyzed charring [101,102]. However, the application of multiple FRs can also lead to competition between the ongoing processes and cause the opposite effect, e.g., the destruction of the protective layer.

Most materials, except PU with 10 wt% of unmodified GTR, were characterized by the TTI of 8 ± 2 s. The cellular structure of PU and low thermal conductivity influence the burning behavior, and TTI usually reaches only a few seconds [103]. Applying more GTR enhanced the time to ignition; however, the use of FRs, whose mechanisms of action force earlier decomposition, eliminated this effect. The comparison of pHRR, an essential parameter to evaluate the burning intensity of PU, confirms the reduction as a consequence of the FR incorporation. The lowest value, approximately four times lower than PU with 5 wt% of GTR, was obtained for the composition GTR_5_FR, containing TCPP and EG. Moreover, along with the GTR share growth, apart from the system with APP and contrary to the samples without FRs, an increase in the analyzed parameter was observed. The reduction due to the FR application was also noted for the MARHE, enabling flame spread evaluation.

A detailed analysis shows that samples containing modified GTR differed more obviously in terms of HRR parameters. MARHE is deduced from the maximum HRR, and in some cases, including the deformation of samples, determining an appropriate value of the index may be problematic [92]. The THR corresponding to the total heat output up to the defined point for GTR_5_ and GTR_10_ was 167 MJ/m^2^ and 150 MJ/m^2^, respectively. The reduction for all investigated samples, from 22 to 36%, may follow from char forming or reduced combustion efficiency [104]. Since the EHC was reduced from 22 MJ/kg (GTR_5_) to 17 MJ/kg (GTR_5_FR and GTR_APP5_FR), both mechanisms of action are possible.

Char formation and dilution of the gas phase significantly affect smoke production. It can be seen that the incorporation of FRs noticeably increased the char yield, which arises from the formation of the protective layer. The reduction of the SEA was recorded for foams with GTR modified by MC (363 m^2^/kg) and MPP (338 m^2^/kg). Unfortunately, increasing the share of GTR from 5 to 10 wt% adversely affected the amount of incomplete combustion products suspended in the gas phase. A similar relationship was observed in the case of TSR. The lowest TSR was observed for foams containing GTR modified with MC and MPP. Considering the amount of heat and smoke released, the most promising results were obtained for materials that, apart from FRs, also contained GTR modified with them (Figure 5), and the most effective was a system with MC. Upon thermal decomposition of MC, melamine is sublimated, whereas cyanuric acid catalyzes chain scission of polymers, causing a decrease in melt viscosity and enhanced dripping, which removes heat from the polymer [96]. The limited dripping during the calorimetric measurements did not have a significant effect, while the released melamine diluted the gas phase effectively.

*Flame Retardancy Index* (*FRI*) is a well-accepted dimensionless index by which polymer composites can be classified based on their flame retardancy performance on a semi-logarithmic scale, such that labels of *Poor*, *Good*, or *Excellent* are defined to be assigned to flame retardant composites having *FRI* values less than 10^0^, between 10^0^ and 10^1^, or above 10^1^, respectively [105]. This index remained powerful and successful in classifying both flame-retardant thermoplastic [106] and thermoset [107] polymer composites. Values of *FRI* of studied PU/GTR composites containing FRs of different family are calculated and summarized with their corresponding labels (Table 4). Two types of flame retardancy performance behavior (label) are observed (*Poor* and *Good*) depending on GTR amount (dominantly controlling factor) and *FR* type (slightly controlling factor). It is also interesting to note that *FRI* varied quite widely in the range of 0.94 to 7.56, which is not the case frequently. It is crystal clear that samples possessing less GTR are less flammable. Moreover, reinforcement of these samples with FRs was more efficiently carried out. Structural change can also be correlated with flame retardancy performance, particularly for GTR_5_FR and GTR_MC5_FR composites with *FRI* values of 7.56 and 4.85, respectively (corresponding to cell size of 202 and 217 μm, according to Table 3). 

## 4. Conclusions

Considering current trends and policies aimed at the enhancement of economic circularity, research works dealing with composites containing recycled or waste-based raw materials are essential. Nevertheless, secondary raw materials, except for the economic and environmental benefits, as well as providing novel functionalities, may carry some burdens affecting the performance of final materials. GTR applied in the presented work may enhance the mechanical and damping performance due to the exceptional properties of car tires, but simultaneously, it is very susceptible to fire. Combining it with PU brings the privilege of PU/GTR composites for applications in the construction and building sector; however, their flammability is a challenge. Therefore, the fire resistance of the resulting PU/GTR composites should be taken into consideration in serious standardized flame retardancy analyses. Moreover, due to the high sensitivity of PU foams and their structure to formulation changes, the introduction of fillers or additives has to be comprehensively investigated in view of microstructural evolutions. The presented study provided vital insights into the structure and performance of PU/GTR composite foams containing GTR particles hybridized with solid FRs, aligning with the current trends inducing application of recycled and waste-based materials, as well as with the tightening fire protection requirements for materials potentially applied in buildings. Changes in the cellular structure, mechanical properties, and thermal insulation properties, often determining the application range of PU foams, were highlighted. *Flame Retardancy Index* (*FRI*)-based classification of composites alongside qualitative analysis of flame retardancy performance revealed helpful information about the role of FRs on a pretty wide range of variation from 0.94 to 7.56 corresponding to *Poor* and *Good* performance labels, respectively.

The multifaceted approach served for flammability reduction, and juggling with the loadings of particular FR additives was also a signature of the possibility of achieving engineered composites to fulfill the requirements of applications. Nevertheless, it is vital to keep in mind that the flammability reduction of PU/GTR composite foams may be achieved at the expense of mechanical and thermal insulation performance. Therefore, future work in the field should focus on masking the performance gap resulting from the introduction of FR without sacrificing fire resistance. Among the potential solutions could be mentioned nanoadditives like clays or proper polyols characterized by the inherent fire resistance related to embedded phosphorous and nitrogen-containing moieties.

## Figures and Tables

**Table 1:** Raw materials applied during manufacturing of polyurethane (PU)/ground tire rubber (GTR) composite foams.

Material	Properties/Additional Information	Producer
Rokopol^®^ F3000	Polyether polyol, hydroxyl value—53–59 mg KOH/g	PCC Group (Brzeg Dolny, Poland)
Rokopol^®^ V700	Polyether polyol, hydroxyl value—225–250 mg KOH/g	PCC Group (Brzeg Dolny, Poland)
Glycerol	Hydroxyl value—1800 mg KOH/g	Sigma Aldrich (Poznań, Poland)
SPECFLEX NF 434	Polymeric methylenediphenyl-4,4′-diisocyanate (pMDI), free isocyanate content—29.5%	M. B. Market Ltd. (Baniocha, Poland)
PC CAT^®^ TKA30 (KAc)	Potassium acetate catalyst	Performance Chemicals (Belvedere, UK)
DABCO 33LV (DABCO)	Catalyst, 33 wt% solution of 1,4-diazabicyclo[2.2.2]octane in dipropylene glycol	Air Products (Allentown, PA, USA)
Dibutyltin dilaurate (DBTD)	Organic tin catalyst	Sigma Aldrich (Poznań, Poland)
Distilled water	Chemical blowing agent	-
Ground tire rubber	Filler	Recykl S.A. (Śrem, Poland)
Expanded graphite	Flame retardant	Nordmann, Rassmann, GmbH (Hamburg, Germany)
Roflam P LO (TCPP)	Flame retardant	PCC Group (Brzeg Dolny, Poland)
Addforce FR MPP (MPP)	Flame retardant, melamine polyphosphate	WTH Walter Thieme Handel GmbH (Stade, Germany)
Budit 314 (MC)	Flame retardant, melamine cyanurate	Chemische Fabrik Budenheim KG (Budenheim, Germany)
Addforce FR APP103 (APP)	Flame retardant, ammonium polyphosphate	WTH Walter Thieme Handel GmbH (Stade, Germany)

**Table 2:** Formulations applied during manufacturing of PU/GTR composite foams.

Component	Foam Symbol					
	GTR_5_	GTR_10_	GTR_5_FR	GTR_10_FR	GTR_X5_FR	GTR_X10_FR
F3000	59.7	59.7	49.75	49.75	49.75	49.75
V700	59.7	59.7	49.75	49.75	49.75	49.75
Glycerol	1.44	1.44	1.20	1.20	1.20	1.20
DBTD	1.08	1.08	0.90	0.90	0.90	0.90
DABCO	0.72	0.72	0.60	0.60	0.60	0.60
KAc	0.72	0.72	0.60	0.60	0.60	0.60
Water	0.63	0.63	0.50	0.50	0.50	0.50
pMDI	56.04	56.04	47.03	47.03	47.03	47.03
TCPP	-	-	14.4	14.4	14.4	14.4
EG	-	-	14.4	14.4	14.4	14.4
GTR/modified GTR	8.85	17.7	7.2	14.4	7.2	14.4

**Table 3:** Parameters of cellular structure, thermal insulation, and mechanical properties of prepared PU/GTR composite foams.

Sample	Cell Size, µm	Circularity	Aspect Ratio	Roundness	Open Cell Content, %	λ Coefficient, mW/(m·K)	Tensile Strength, kPa	Elongation at Break, %
GTR_5_	248 ± 69	0.48 ± 0.23	1.39 ± 0.16	0.73 ± 0.08	80.8 ± 0.7	62.1 ± 2.4	268.9 ± 7.9	116.2 ± 4.4
GTR_10_	209 ± 50	0.48 ± 0.21	1.32 ± 0.15	0.77 ± 0.08	80.7 ± 1.9	57.1 ± 2.5	202.4 ± 9.1	101.7 ± 5.8
GTR_5_FR	202 ± 36	0.54 ± 0.20	1.30 ± 0.17	0.78 ± 0.09	83.7 ± 0.4	68.4 ± 2.4	105.2 ± 3.1	80.2 ± 4.4
GTR_10_FR	223 ± 67	0.47 ± 0.21	1.35 ± 0.17	0.75 ± 0.09	82.0 ± 1.0	71.9 ± 2.0	114.5 ± 4.0	82.6 ± 6.7
GTR_APP5_FR	226 ± 47	0.46 ± 0.20	1.34 ± 0.13	0.75 ± 0.07	82.4 ± 1.4	79.3 ± 2.5	145.6 ± 2.1	82.2 ± 3.6
GTR_APP10_FR	233 ± 74	0.41 ± 0.25	1.37 ± 0.14	0.74 ± 0.08	83.8 ± 3.6	78.9 ± 1.5	129.0 ± 4.5	80.4 ± 5.9
GTR_MC5_FR	217 ± 59	0.48 ± 0.21	1.32 ± 0.11	0.76 ± 0.06	84.7 ± 2.5	75.6 ± 2.3	125.3 ± 5.2	72.8 ± 5.2
GTR_MC10_FR	228 ± 54	0.46 ± 0.21	1.34 ± 0.16	0.75 ± 0.08	85.6 ± 0.7	76.4 ± 1.5	112.9 ± 4.5	66.2 ± 5.2
GTR_MPP5_FR	234 ± 61	0.48 ± 0.22	1.35 ± 0.14	0.75 ± 0.08	85.1 ± 1.0	76.6 ± 2.2	136.8 ± 7.2	73.8 ± 2.8
GTR_MPP10_FR	234 ± 58	0.50 ± 0.18	1.35 ± 0.12	0.75 ± 0.06	83.3 ± 3.6	72.5 ± 2.1	121.3 ± 2.7	68.0 ± 4.7

**Table 4:** Values of limiting oxygen index (LOI) and results of cone calorimeter evaluation of prepared PU/GTR composite foams.

Sample	LOI, %*v*/*v*	TTI, s	pHRR, kW/m^2^	MARHE, kW/m^2^	THR, MJ/m^2^	EHC, MJ/kg	SEA, m^2^/kg	TSR, m^2^/m^2^	Char Yield, %	*FRI* (Label)
GTR_5_	20.0	8 ± 3	461 ± 20	279 ± 33	167 ± 2	22 ± 0	380 ± 19	2906 ± 111	9.9 ± 1.2	*RS
GTR_10_	19.4	21 ± 1	368 ± 49	208 ± 45	150 ± 16	19 ± 3	409 ± 6	3230 ± 116	11.5 ± 0.9	*RS*
GTR_5_FR	28.2	10 ± 5	119 ± 18	95 ± 0	107 ± 5	17 ± 1	431 ± 21	2839 ± 251	26.4 ± 0.6	7.56 (*Good*)
GTR_10_FR	26.6	6 ± 0	144 ± 2	112 ± 20	113 ± 6	19 ± 2	484 ± 16	3390 ± 458	27.8 ± 0.1	0.97 (*Poor*)
GTR_APP5_FR	27.0	9 ± 0	187 ± 2	114 ± 1	108 ± 1	17 ± 1	409 ± 10	2571 ± 68	26.8 ± 0.0	4.29 (*Good*)
GTR_APP10_FR	26.2	7 ± 1	154 ± 5	127 ± 1	124 ± 3	19 ± 1	486 ± 12	3173 ± 84	27.3 ± 0.0	0.96 (*Poor*)
GTR_MC5_FR	28.0	8 ± 1	138 ± 20	142 ± 25	115 ± 0	18 ± 0	363 ± 36	2297 ± 162	27.5 ± 0.2	4.85 (*Good*)
GTR_MC10_FR	26.4	9 ± 0	167 ± 36	117 ± 5	114 ± 0	19 ± 0	380 ± 5	2665 ± 461	28.2 ± 05	1.24 (*Good*)
GTR_MPP5_FR	29.4	7 ± 3	165 ± 38	132 ± 14	122 ± 4	19 ± 0	338 ± 9	2250 ± 228	25.2 ± 1.3	3.35 (*Good*)
GTR_MPP10_FR	27.6	9 ± 0	191 ± 15	129 ± 5	131 ± 10	20 ± 2	397 ± 117	2606 ± 829	24.9 ± 1.7	0.94 (*Poor*)

*RS: Reference Samples, which have not taken FRI values.

## Data Availability

Data are contained within the article.

## Supplementary Materials

The following supporting information can be downloaded at: https://www.mdpi.com/article/10.3390/polym16050656/s1, Figure S1: SEM images showing the appearance of the surface of GTR particles applied in the presented study.
