# Supplementary material for: Fire-Retardant Flexible Foamed Polyurethane (PU)-Based Composites: Armed and Charmed Ground Tire Rubber (GTR) Particles
Source: Polymers (Basel). 2024 Feb 28;16(5):656. doi: 10.3390/polym16050656 (PMC10934310; doi:10.3390/polym16050656)
Supplement: Supplementary file 1 [file polymers-16-00656-s001.zip › polymers-2862322-supplementary.pdf]

polymers

MDPI

Supplementary Materials

Fire-Retardant Flexible Foamed Polyurethane (PU)-Based Composites: Armed and Charmed Ground Tire Rubber (GTR) Particles

Paulina Kosmela [1], Kamila Sałasińska [2], Daria Kowalkowska-Zedler [3], Mateusz Barczewski [4], Adam Piasecki [5], Mohammad Reza Saeb [6] and Aleksander Hejna [1,4,*]

[1] Department of Polymer Technology, Gdańsk University of Technology, Narutowicza 11/12, 80-233 Gdańsk, Poland; paulina.kosmela@pg.edu.pl
[2] Faculty of Materials Science and Engineering, Warsaw University of Technology, Wołoska 141, 02-507 Warsaw, Poland; kamila.salasinska@pw.edu.pl
[3] Department of Inorganic Chemistry, Gdańsk University of Technology, Narutowicza 11/12, 80-233 Gdańsk, Poland; daria.zedler@pg.edu.pl
[4] Institute of Materials Technology, Poznan University of Technology, Piotrowo 3, 61-138 Poznań, Poland; mateusz.barczewski@put.poznan.pl
[5] Institute of Materials Engineering, Poznan University of Technology, Jana Pawła II 24, 60-965 Poznan, Poland; adam.piasecki@put.poznan.pl
[6] Department of Pharmaceutical Chemistry, Medical University of Gdańsk, J. Hallera 107, 80-416 Gdańsk, Poland; mrsaeb2008@gmail.com
* Correspondence: aleksander.hejna@put.poznan.pl

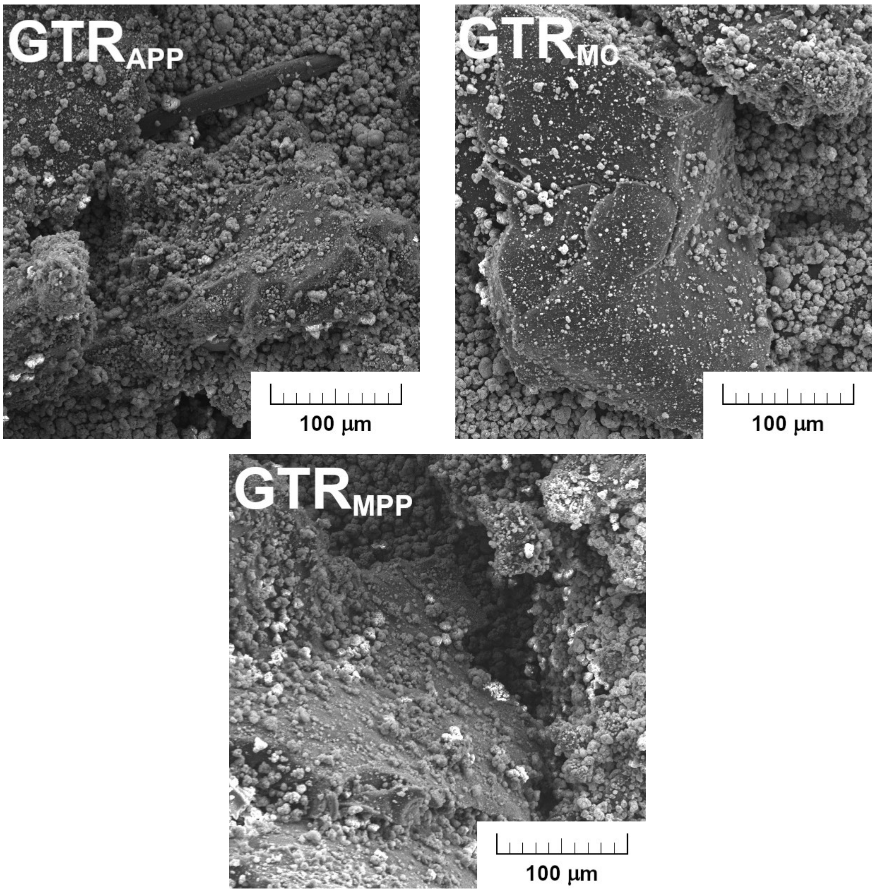

Figure S1. SEM images showing the appearance of the surface of GTR particles applied in the presented study.
